# Poisonous Expectoration in Phthisis Pulmonalis

**Published:** 1828-08

**Authors:** 


3. *Poisonous Expectoration in Phthisis.*—The following fact, although not of recent date, deserves to be recorded.

In the year 1802, Judge McMillan, of the neighborhood of this city, a few weeks before his death, from Phthisis Pulmonalis, informed his physician, Dr. Goforth, that when sitting at his door, and accustomed as he was to spit the matter which he coughed up on the surrounding turf, he frequently saw it eaten by chickens, in whom it produced *vestigo terminating in a transient state of insensibility and loss of muscular power.* The intelligence and veracity of both the patient and physician, were such, that we see no reason to doubt the fact.

